# Research on podocyte injury mechanisms in diabetic nephropathy: a bibliometric and knowledge-map analysis from 2000 to 2024

**DOI:** 10.3389/fendo.2025.1578045

**Published:** 2025-08-11

**Authors:** Yanmei Lin, Jianqing Tian

**Affiliations:** Fujian Medical University Xiamen Humanity Hospital, Xiamen, Fujian, China

**Keywords:** VOSviewer, CiteSpace, bibliometrics analysis, bibliometrix, podocyte injury, DN

## Abstract

**Objectives:**

A bibliometric and knowledge-map analysis is used to explore hotspots evolution and development trends in the Podocyte Injury Mechanisms in Diabetic Nephropathy. By looking for research hotspots and new topics, we can provide new clues and ideas for researchers in this field.

**Methods:**

The articles and reviews regarding Podocyte were retrieved and obtained from the Web of Science Core Collection (WOSCC) on September 1st, 2024. CtieSpace [version 6.2.4R (64-bit)] and VOSviewer (version 1.6.18) were used to conduct the bibliometric and knowledge-map analysis.

**Results:**

12086 authors from 2394 institutions in 69 countries/regions published 3239 papers in academic journals. PEOPLES R CHINA and THE USA were absolutely in the leading position in this research field. The institution that contributed the most publications was the Shandong University. Kidney International (130 articles) is the journal with the most published records and the highest number of co-citations. The author with the highest number of co citations is Mundel p. However, there was little cooperation between countries. After 2012, cooperation among various institutions was also small. Autophagy, mitochondria, and epigenetics are hot topics and trends in this field. The most influential research hotspots were the research of podocyte autophagy and metabolism, the related research of SIRT1, VEGF. The latest hotspots and topics included the study of nlrp3 inflammasome and signaling pathway in podocyte injury. The research of Podocyte in Diabetic Nephropathy was a rapidly developing hot field.

**Conclusion:**

The damage mechanism of podocytes is a very important factor in the occurrence and progress of diabetes nephropathy. Early intervention of podocyte damage is a very potential preventive measure for diabetes nephropathy, which has clinical application prospects and is currently being rapidly developed.

## Introduction

Diabetic nephropathy (DN), a common and serious microvascular complication of diabetes, is the leading cause of end-stage renal disease (ESRD) and is associated with increased risks of cardiovascular disease and death in high-risk individuals. Up to 40% of people with diabetes will develop chronic kidney disease (CKD) (1, 2).

The kidneys filter approximately 180 L of fluids everyday; however, there is no loss of proteins into urine as the glomerular filtration barrier (GFB) retains 99.99% of plasma proteins. Alterations in the GFB result in the development of proteinuria, ranging from albuminuria to massive nephrotic syndrome.

Podocytes are highly specialized glomerular epithelial cells that, along with the fenestrated endothelium and the glomerular basement membrane (GBM), collectively form the glomerular filtration barrier (GFB). The podocyte cell body protrudes into the urinary space and gives rise to elongated primary processes, which branch into foot processes (FPs) that envelop the glomerular capillaries. Podocyte dysfunction is implicated, either primarily or secondarily, in all forms of proteinuric glomerular diseases, as evidenced by the morphological alterations in their intricate cellular architecture detectable through electron microscopy. Hence, recent research has increasingly focused on the role of podocyte injury in DN, with a growing emphasis on exploring therapeutic interventions targeting podocyte injury (1). Studies have revealed that factors such as lipotoxicity, hemodynamic abnormalities, oxidative stress, mitochondrial dysfunction, and impaired autophagy can contribute to podocyte injury. Consequently, the purpose of this bibliometric analysis was to summarize the underlying mechanisms of podocyte injury in DN and provide an overview of the current research status regarding experimental drugs targeting podocyte injury in DN. The findings presented herein may offer potential therapeutic targets and strategies for the management of DN associated with podocyte injury.

## Materials and methods

### Database selection

The Web of Science Core Collection (WoSCC) database was chosen for this study due to its superior accuracy in annotating literature types compared to other databases, making it the optimal choice for comprehensive literature analysis. WoSCC’s rigorous indexing and citation tracking capabilities ensure high-quality data for bibliometric studies, which was crucial for our investigation into podocyte research in diabetic nephropathy.

### Search strategy

On September 1, 2024, we conducted a comprehensive search in the WoSCC database to retrieve all articles related to podocyte research in diabetic nephropathy from January 1, 2000, to September 1, 2024. The search was designed to capture a broad spectrum of relevant literature while maintaining specificity. The search formula employed was as follows:TS=(Podocytes) OR TS=(Podocyte) AND (((((((((((((((((TS=(“Diabetic Nephropathies”)) OR TS=(“Nephropathies, Diabetic”)) OR TS=(“Nephropathy, Diabetic”)) OR TS=(“Diabetic Nephropathy”)) OR TS=(“Diabetic Kidney Disease”)) OR TS=(“Diabetic Kidney Diseases”)) OR TS=(“Kidney Disease, Diabetic”)) OR TS=(“Kidney Diseases, Diabetic”)) OR TS=(“Diabetic Glomerulosclerosis”)) OR TS=(“Glomerulosclerosis, Diabetic”)) OR TS=(“Intracapillary Glomerulosclerosis”)) OR TS=(“Nodular Glomerulosclerosis”)) OR TS=(“Glomerulosclerosis, Nodular”)) OR TS=(“Kimmelstiel-Wilson Syndrome”)) OR TS=(“Kimmelstiel Wilson Syndrome”)) OR TS=(“Syndrome, Kimmelstiel-Wilson”)) OR TS=(“Kimmelstiel-Wilson Disease”)) OR TS=(“Kimmelstiel Wilson Disease”).This formula combined keywords related to podocytes with various terms for diabetic nephropathy and its associated conditions to ensure comprehensive coverage of the topic.

### Duplicate management

After the initial search, duplicates were identified and removed using a two-step process. First, automated tools were employed to flag potential duplicates based on title, abstract, and full-text similarities. Subsequently, a manual review was conducted by two independent reviewers to confirm and remove any remaining duplicates, ensuring the accuracy and integrity of the dataset used for analysis.

### Inclusion and exclusion criteria

The literature selection for this study was based on the following inclusion criteria:

1. Full-text Publications: Articles must have full-text availability focusing on podocytes in the context of diabetic nephropathy.
2. Language: Both articles and review manuscripts must be written in English to ensure consistency and ease of analysis.

The exclusion criteria were as follows:

1. Irrelevant Theme: Articles not directly related to podocytes in diabetic nephropathy were excluded.
2. Non-Research Formats: Conference abstracts, news articles, briefings, and other non-research formats were excluded to focus on peer-reviewed research.

### Screening process and inter-reviewer reliability

To ensure the rigor and transparency of the screening process, a second reviewer was involved in addition to the primary reviewer. Both reviewers independently screened the titles and abstracts of the retrieved articles based on the inclusion and exclusion criteria. Any discrepancies between the two reviewers were resolved through discussion and consensus. To further enhance transparency, we calculated the inter-reviewer agreement rate, which was found to be high (e.g., Cohen’s Kappa coefficient > 0.8), indicating excellent agreement between the reviewers.

### Data export

The final dataset, after applying the inclusion and exclusion criteria and removing duplicates, was exported in a pure text version for further analysis (3, 4).

### Date collection

Author names, nationalities and affiliations, article title, year of publication, name of publishing journal, keywords, and abstract were collected from eligible articles. The WoSCC was used to download all records as a.txt file.

### Bibliometric analysis

Use Graphpad prism v8.0.2 to analyze and plot annual and national publication trends and ratios. In addition, CtieSpace (6.2.4R (64 bit) advanced version) and VOSviewer (1.6.18 version) were used to analyze these data and visualize the scientific knowledge graph.

VOSviewer v. 1.6.18 was created by Waltman et al. in 2009 and is a free software based on JAVA used to analyze large amounts of literature data and display it in map format. In order to visualize research results in a certain field by drawing a literature co citation network diagram, Professor Chen Chaomei created CiteSpace (6.2.4R) software, which envisions using an experimental framework to study new concepts and evaluate existing technologies. This enables users to better understand the knowledge field, research frontiers, and trends, and predict their future research progress (3, 4).

## Result

### Publication outputs and time trend

**Figure 1:**
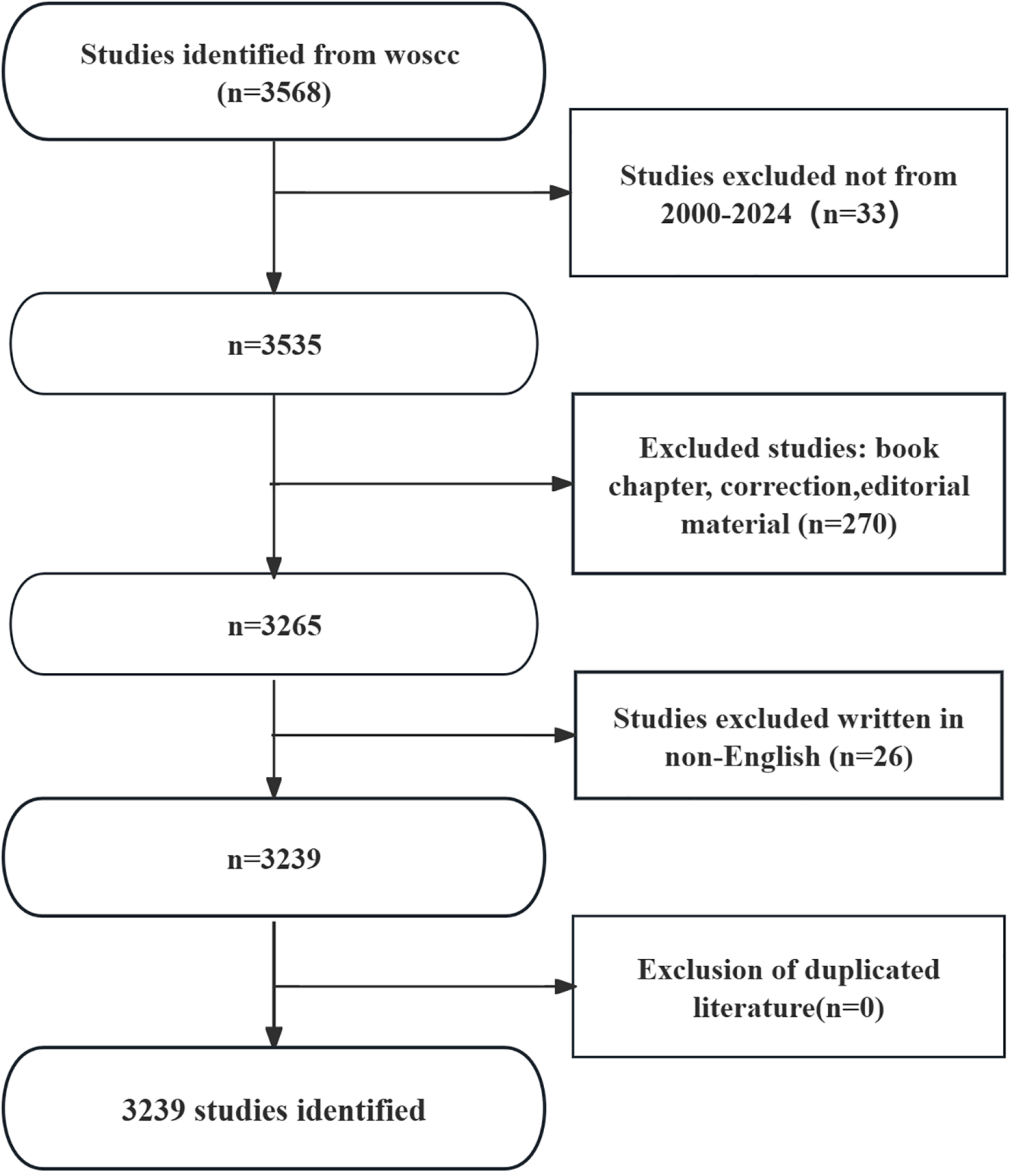
Flowchart of literature search.

The results showed that there were 3239 articles (**Figure 1**) related to podocyte in diabetes nephropathy research in WoSCC database, including 993 articles and 370 reviews. The literature covers 69 countries and regions, 2394 institutions, and 12086 authors.

**Figure 2:**
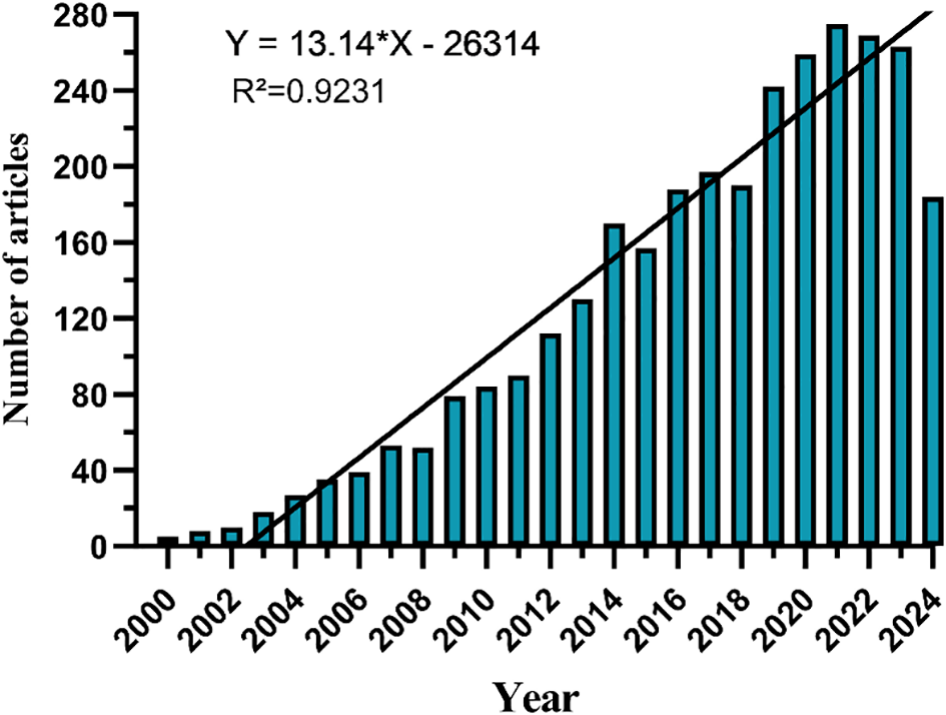
Annual volume of publications.

Since 2000, the number of records published each year has been slowly increasing. We divide it into three stages. From 2000 to 2006, the growth was slow (**Figure 2**), with an annual publication volume of less than 50 articles, indicating slow development in this field. From 2007 to 2013, the publication volume increased rapidly, and after 2014, the growth rate of publication volume further increased and reached its highest point in 2021.

### Distribution of country and institution

**Figure 3:**
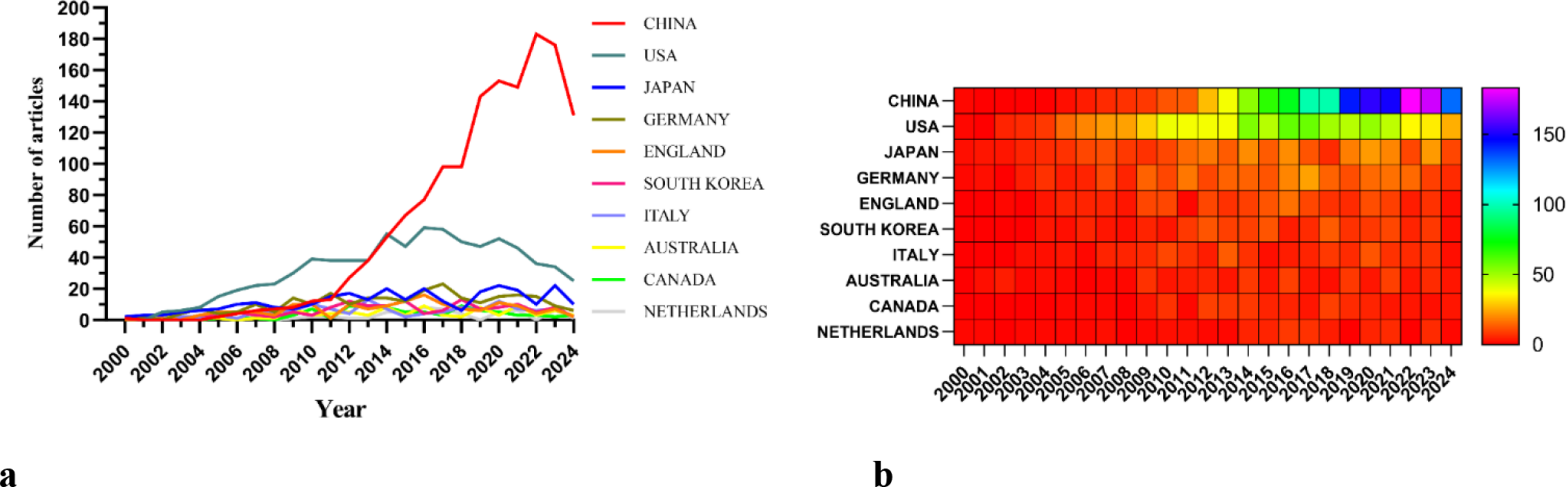
**(a)** Line graph of national publications. **(b)** Heat map of national publications.

There are 69 countries and regions that have conducted research on the application of podocytes in diabetic nephropathy research. **Figures 3** illustrate the annual publication volume of the top 10 countries over the past decade. Among these, the top five countries in this field are China, the United States, Japan, Germany, and the United Kingdom. China’s publication volume accounts for 44.64% of the total, significantly surpassing other countries.

Among the top ten countries/regions in terms of publication volume, the United States’ records have been cited 49,163 times (**Table 1**), far exceeding all other countries/regions. Its paper citation-to-publication ratio (62.23) ranks third among all countries, indicating that the quality of its published papers is generally high. China ranks first in terms of publication volume (1,446), second in terms of citation frequency (36,621), but has a relatively low citation-to-publication ratio (25.33).

**Table 1:** Table of country published literature.

Rank	Country/region	Article counts	Centrality	Percentage (%)	Citation	Citation per publication
1	CHINA	1446	0.13	44.64%	36621	25.33
2	USA	790	0.46	24.39%	49163	62.23
3	JAPAN	289	0.12	8.92%	12780	44.22
4	GERMANY	257	0.17	7.93%	16871	65.65
5	ENGLAND	153	0.14	4.72%	8127	53.12
6	SOUTH KOREA	134	0.03	4.14%	4496	33.55
7	ITALY	124	0.11	3.83%	7443	60.02
8	AUSTRALIA	91	0.06	2.81%	5722	62.88
9	CANADA	83	0.02	2.56%	4587	55.27
10	NETHERLANDS	62	0.12	1.91%	3708	59.81

**Figure 4:**
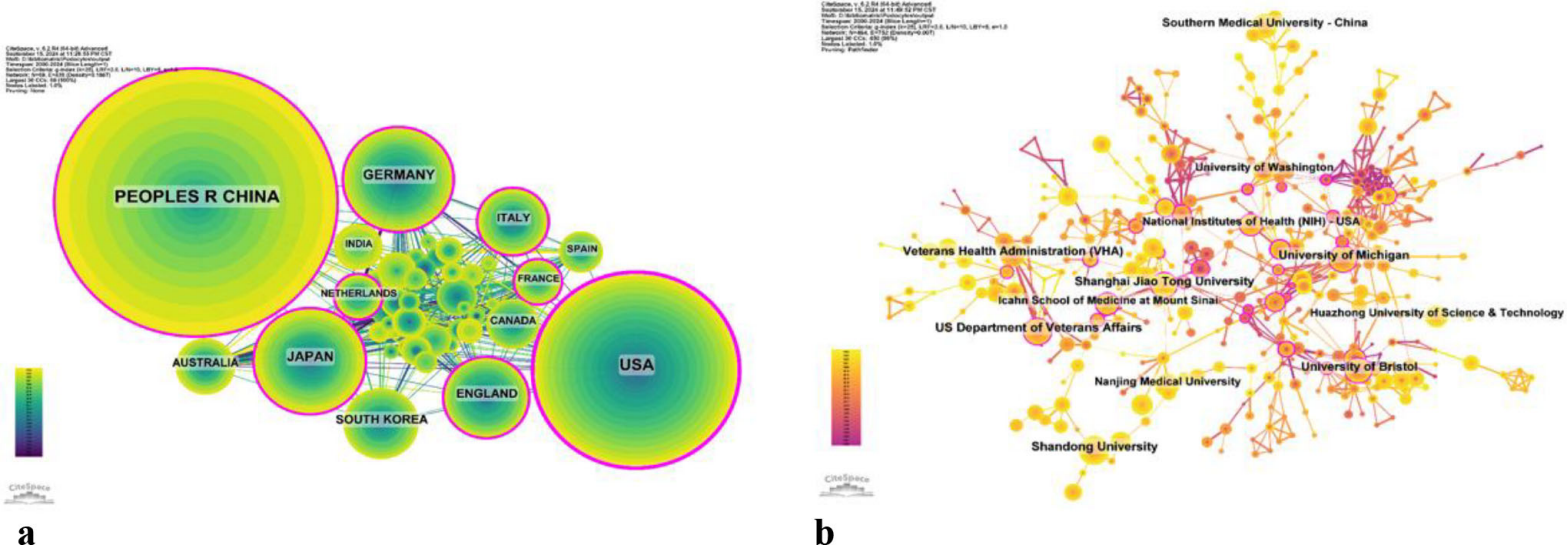
**(a)** Networks of country cooperation. **(b)** Networks of institutional co-operation.

The cooperation network is depicted in **Figure 4a**. The United States has close cooperation with countries such as the United Kingdom, France, and Canada, while China has closer ties with countries like Germany, Japan, and South Korea. China not only boasts a substantial number of publications and a high citation frequency but also has a centrality of 0.13, suggesting that it is currently a leading country in this field.

2394 institutions systematically published articles related to podocyte research in diabetes nephropathy. Among the top ten institutions in terms of publication volume, 5 are from the United States, 4 are from China, and 1 is from the United Kingdom (**Table 2**, **Figure 4b**). Shandong University has the highest number of publications (86 papers, 2360 citations, 27.44 citations per paper). The US Department of Veterans Affairs (80 papers, 5703 citations, 71.29 citations/paper) ranks second, the University of Michigan (77 papers, 7253 citations, 94.19 citations/paper) ranks third, and the Veterans Health Administration (VHA) (74 papers, 5488 citations, 74.16 citations/paper) ranks fourth. After further analysis, we found that domestic and foreign institutions are more inclined to cooperate with their own domestic units. Therefore, we call for strengthening cooperation between domestic and foreign institutions and breaking down academic barriers.

**Table 2:** Table of institutional published literature.

Rank	Institution	Country	Number of studies	Total citations	Average citation
1	Shandong University	China	86	2360	27.44
2	US Department of Veterans Affairs	USA	80	5703	71.29
3	University of Michigan	USA	77	7253	94.19
4	Veterans Health Administration (VHA)	USA	74	5488	74.16
5	University of Bristol	England	73	4481	61.38
6	Shanghai Jiao Tong University	China	70	2483	35.47
7	Southern Medical University - China	China	65	1519	23.37
8	Huazhong University of Science & Technology	China	57	2594	45.51
9	Icahn School of Medicine at Mount Sinai	USA	54	3544	65.63
10	National Institutes of Health (NIH) - USA	USA	54	4572	84.67

### Distribution of journal and research area

**Tables 3**, **4** present the top 10 journals with the highest output and most citations in the field. Kidney International stands out as the journal with the most published records in this domain, featuring 130 articles, accounting for 4.01% of the total. It is followed closely by American Journal of Physiology: Renal Physiology with 126 articles (3.89%), Journal of the American Society of Nephrology with 113 articles (3.49%), and Diabetes with 70 articles (2.16%). Notably, among the top ten most prolific journals, Kidney International boasts the highest impact factor (IF) of 14.8. Additionally, 90% of these journals are classified within the Q1 region.

**Table 3:** Table of journal publications.

Rank	Journal	Article counts	Percentage(3239)	IF	Quartile in category
1	kidney international	130	4.01%	14.8	Q1
2	american journal of physiology-renal physiology	126	3.89%	3.7	Q1
3	journal of the american society of nephrology	113	3.49%	10.3	Q1
4	diabetes	70	2.16%	6.2	Q1
5	frontiers in pharmacology	69	2.13%	4.4	Q1
6	plos one	69	2.13%	2.9	Q1
7	nephrology dialysis transplantation	68	2.10%	4.5	Q1
8	scientific reports	59	1.82%	3.8	Q1
9	biochemical and biophysical research communications	56	1.73%	2.5	Q3
10	international journal of molecular sciences	51	1.57%	4.9	Q1

**Table 4:** Co-citation table of journals.

Rank	Cited journal	Co-citation	IF (2023)	Quartile in category
1	KIDNEY INT	2741	14.8	Q1
2	J AM SOC NEPHROL	2688	10.3	Q2
3	DIABETES	2223	6.2	Q1
4	AM J PHYSIOL-RENAL	2125	3.7	Q1
5	J CLIN INVEST	2027	13.3	Q1
6	J BIOL CHEM	1687	4.0	Q2
7	NEPHROL DIAL TRANSPL	1553	4.8	Q1
8	DIABETOLOGIA	1423	8.4	Q1
9	PLOS ONE	1394	2.9	Q1
10	AM J PATHOL	1328	4.7	Q1

**Figure 5:**
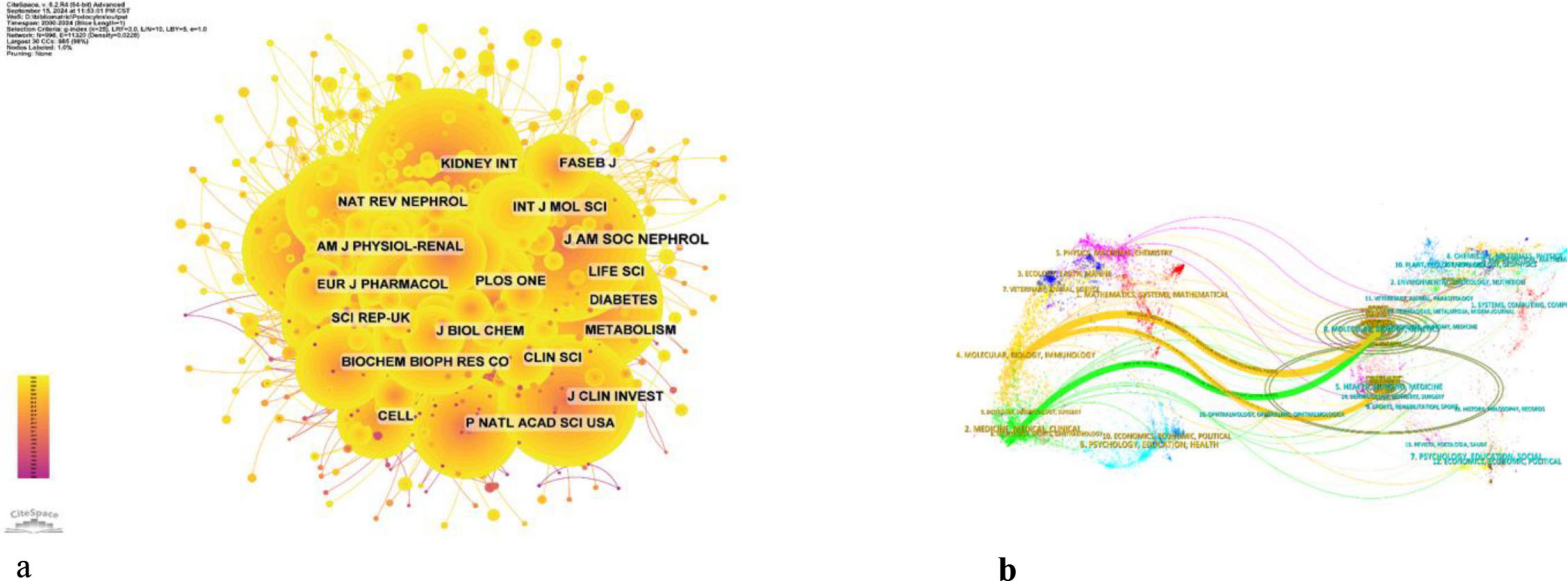
**(a)** Co-citation network map of journals. **(b)** Dual map of journals.

The influence of a journal is gauged by its frequency of co-citation, serving as an indicator of its substantial impact on the scientific community. As illustrated in **Figure 5** and **Table 4**, KIDNEY INT emerges as the journal with the highest number of co-citations, totaling 2741 times. It is succeeded by J AM SOC NEPHROL with 2688 co-citations and DIABETES with 2223 co-citations. Within the top 10 journals that have been jointly cited the most, KIDNEY INT not only stands out with its 2741 co-citations but also possesses the highest impact factor (IF) of 14.8 among the top 10. All of the jointly cited journals are situated within the Q1 or Q2 regions.

**Figure 6:**
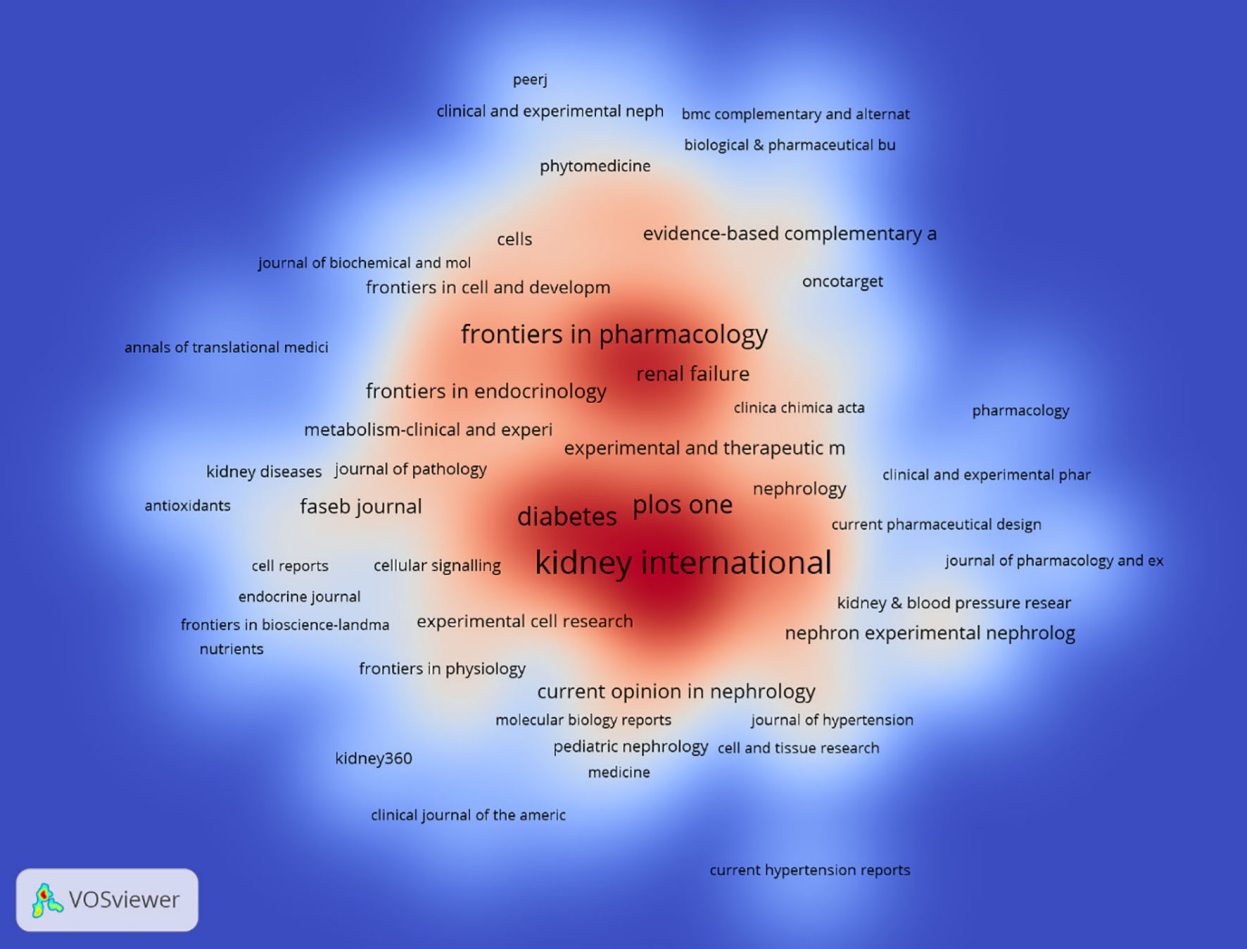
Density map of journal publications.

The thematic distribution of academic publications is displayed through a dual map overlay (**Figure 6**). The colored trajectory represents citation connections, with the cited journal on the left and the cited journal on the right. Based on the displayed results, we have identified two main color citation pathways, where research published in journals in the fields of molecular/biology/genetics is mainly cited by research published in journals in the fields of molecular/biology/immunology and medicine/medical/clinical. The research published in journals in the field of health/oncology/medicine is mainly cited by research published in journals in the field of molecular/biology/immunology.

### Analysis of authors

**Figure 7:**
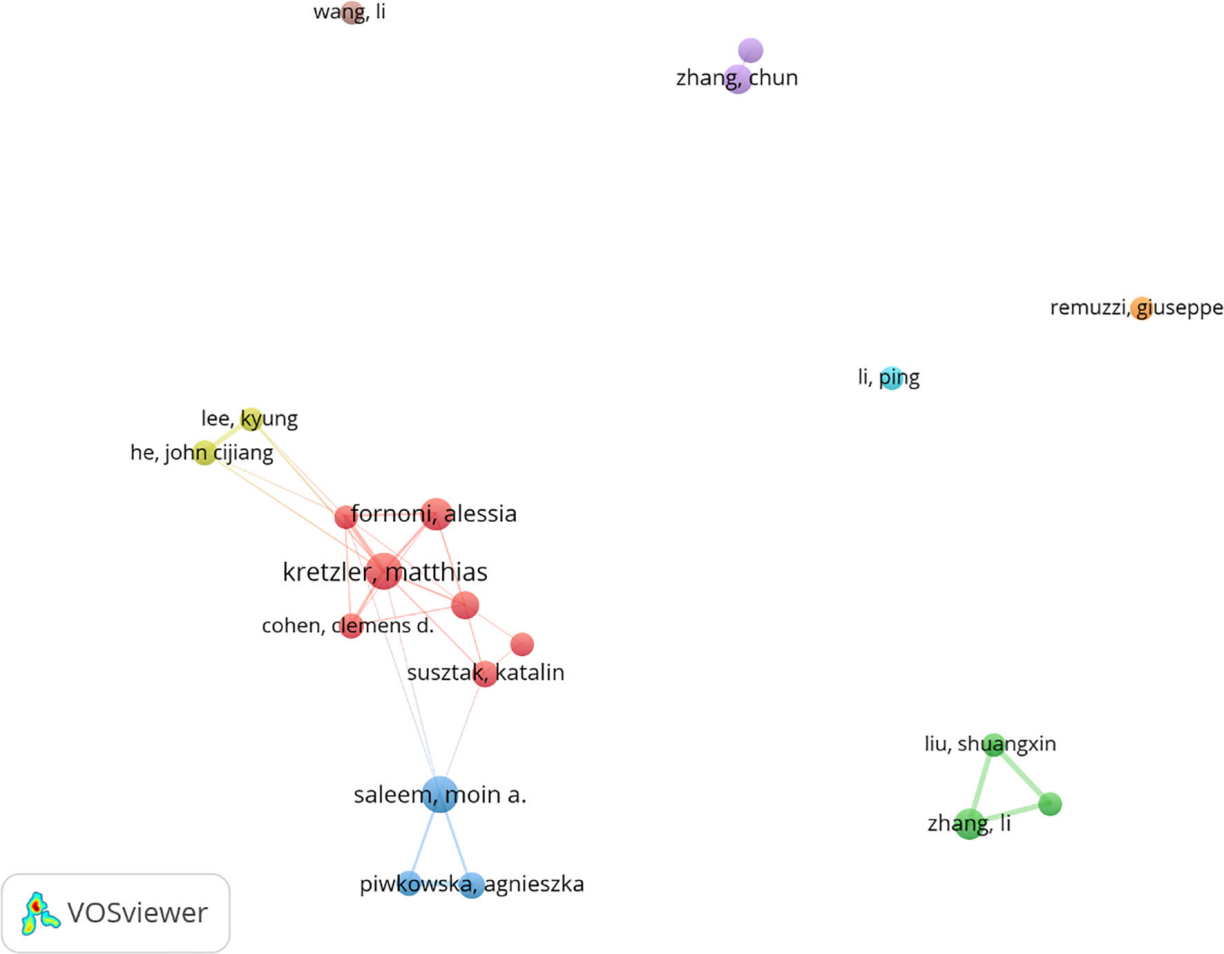
Cooperation network of authors.

Among all the authors who have published relevant literature on podocyte in diabetes nephropathy research, **Table 5** lists the 10 authors who have published the most papers. The top 10 authors have published a total of 280 papers, accounting for 8.58% of all papers in the field. Kretzler and Matthias have published the most research papers, with 37 papers, followed by Saleem and Moin a. (36 papers) and Fornoni and Alesia (31 papers). CiteSpace visualizes the network between authors (**Figure 7**).

**Table 5:** Author’s publications and co-citation table.

Rank	Author	Count	Rank	Co-cited author	Citation
1	Kretzler, matthias	37	1	Mundel p	527
2	Saleem, moin a.	36	2	Susztak k	466
3	Fornoni, alessia	31	3	Pagtalunan me	460
4	Zhang, li	29	4	Wolf g	432
5	Zhang, chun	28	5	Shankland sj	341
6	Huber, tobias b.	26	6	Kriz w	307
7	Piwkowska, agnieszka	24	7	Saleem ma	284
8	Susztak, katalin	24	8	Pavenstadt h	281
9	Zhang, yan	23	9	Sharma k	275
10	Cohen, clemens d.	22	10	Reiser j	265

**Figure 8:**
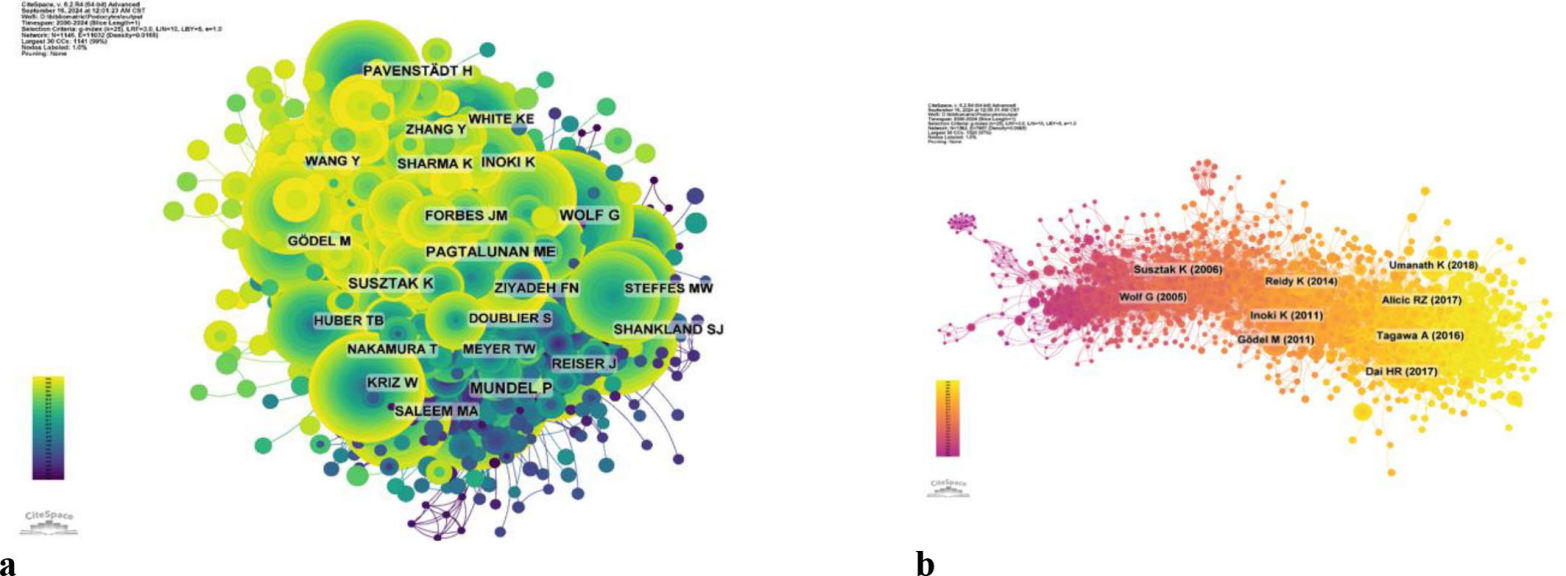
**(a)** Co-citation network of authors. **(b)** Co-cited network of literature.

**Figure 8** and **Table 5** respectively display the top 10 authors who have been co cited and cited the most times. 194 authors have been cited over 50 times, indicating the high reputation and influence of their research. The largest nodes are associated with the authors with the highest number of co citations, including Mundel p (527 citations), Susztak k (466 citations), and Pagtalunan me (460 citations).

### Analysis of keywords

**Figure 9:**
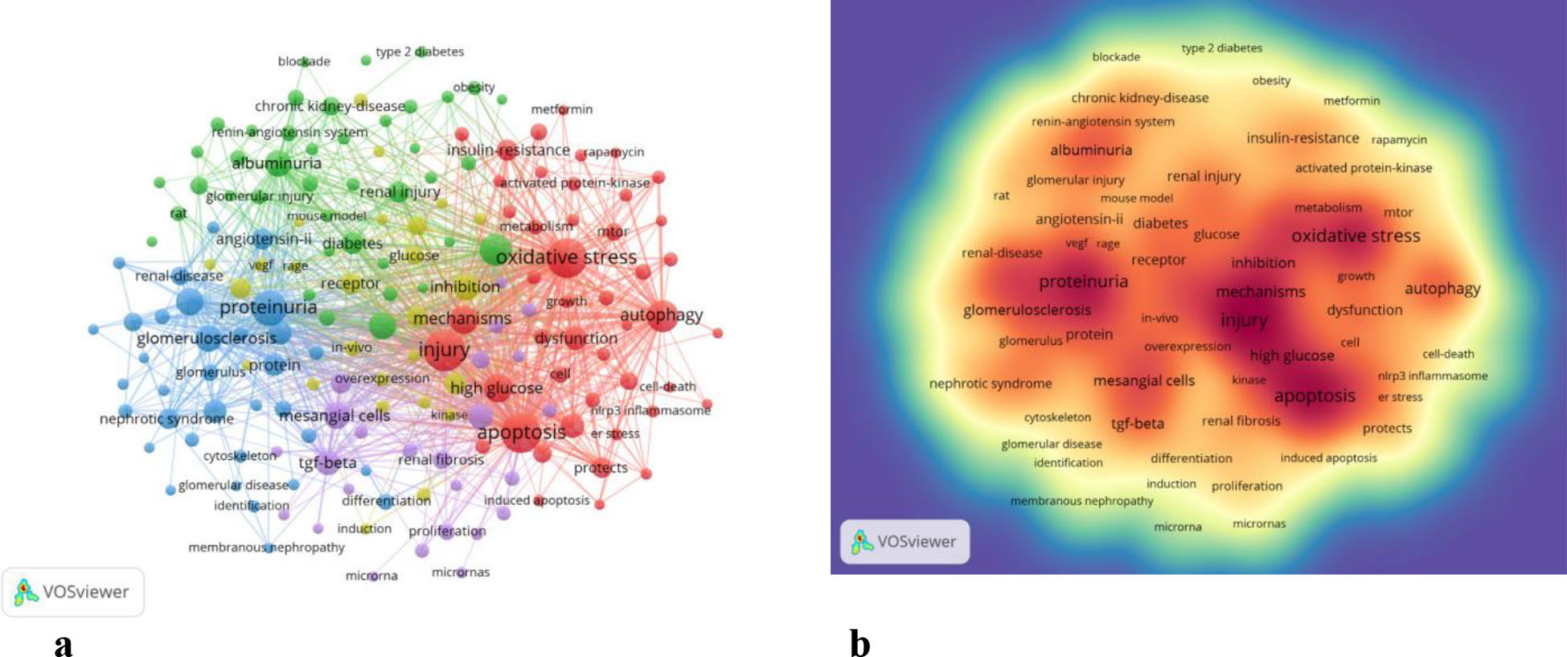
**(a)** Network map of high-frequency. **(b)** Density map of keywords.

By analyzing keywords, we swiftly grasp the status and development trajectory of a field. According to VOSviewer’s keyword co-occurrence analysis (**Table 6**, **Figure 9**), “injury” (517 mentions) tops the list, followed by “apoptosis” (474), “oxidative stress” (462), “proteinuria” (374), and “inflammation” (301). After filtering out irrelevant keywords, we constructed a network encompassing 172 keywords appearing at least 29 times, forming five distinct clusters.

**Table 6:** High frequency keyword table.

Rank	Keyword	Counts	Rank	Keyword	Counts
1	injury	517	11	glomerulosclerosis	203
2	apoptosis	474	12	inhibition	199
3	oxidative stress	462	13	albuminuria	197
4	proteinuria	374	14	mesangial cells	195
5	inflammation	301	15	fibrosis	172
6	autophagy	287	16	tgf-beta	169
7	mechanisms	228	17	pathway	162
8	high glucose	224	18	focal segmental glomerulosclerosis	148
9	nephrin	222	19	gene-expression	146
10	progression	218	20	dysfunction	145

Cluster 1 (red) includes 46 keywords focusing on oxidative stress, autophagy, injury, apoptosis, mechanisms, AMPK, AKT, high glucose metabolism, mTOR, NADPH oxidase, NRF2, NLRP3 inflammasome, and SIRT1. Cluster 2 (green) comprises 41 keywords centered on inflammation progression, diabetes, renal injury, insulin deficiency, blood pressure complications, models, macrophages, risks, streptozotocin, systems, therapies, and obesity.

Cluster 3 (blue) has 34 keywords related to nephrin, proteinuria, proteins, glomerulosclerosis, growth factors, podocalyxin, epithelial cells, glomeruli, IgA nephropathy, mRNAs, mutations, podocin, and slit diaphragms. Cluster 4 (yellow) encompasses 26 keywords dealing with inhibition, pathogenesis, kinases, receptors, glucose, RAGE, VEGF, angiogenesis, nitric oxide, PKC, mouse models, and damage. Cluster 5 (purple) includes 25 keywords centered on mesenchymal cells, renal fibrosis, microRNA, proliferation, biomarkers, acute kidney injury, cancer, high glucose, ILK, NF-κB, TGF-β, and upregulation.

**Figure 10:**
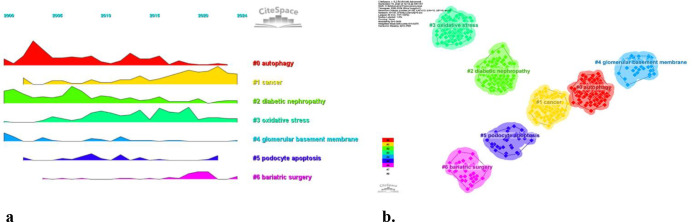
**(a)** Peak map of keyword clustering. **(b)** Clustering map of keywords.

Using CiteSpace, we created a volcano map to visually depict the evolution of research hotspots over time (**Figure 10**). Current research hotspots include autophagy, cancer, oxidative stress, podocyte apoptosis, and bariatric surgery.

## Discussion

In this study, we applied bibliometric methods for the first time to reveal the intellectual bases and emerging trends of podocyte injury and implications for diabetic nephropathy, aiming to inform readers of the recent knowledge and future research directions of this field. In addition, we also introduced journals, institutions, and authors that readers could rely on to obtain knowledge on this subject.

### General trends in the research field from 2000 to 2024

Podocyte injury for diabetic nephropathy has received a growing amount of attention. The total quantity of publications has steadily increased over the past 2 decades.

In addition, it is anticipated that the annual publication output will increase significantly over the next decade, which bodes well for the future of this research field.

China, the United States, Japan and Germany all made significant contributions to the publication of articles. China, the United States, and Japan collaborated with numerous nations in this field. Top five productive institutions accounted for 508 articles. Among them, Shandong University ranked first, followed by US Department of Veterans Affairs and the University of Michigan. China’s large publication volume, high citation frequency, and centrality reaching 0.13 indicate that it is currently a leading country in this field (**Figure 4**, **Table 2**). After further analysis, we found that domestic and foreign institutions are more inclined to cooperate with their own domestic units. Therefore, we call for strengthening cooperation between domestic and foreign institutions and breaking down academic barriers. Top five performing journals published 508 articles, making up 15.68% of all articles. These journals’ impact factors (IF) in 2024 range from 4.4 to 14.8. The Kidney International (IF, 2024 = 14.8), American journal of Physiology-renal Physiology (IF, 2024 = 3.7), Diabetes (IF, 2024 = 6.2)and journal of the American society of Nephrology and (IF, 2024 = 10.3) focus on cell biology; Developmental Biology; Genetics; Cellular and transport physiology; Hemodynamics and vascular regulation; Immunology and Pathology; Pathophysiology of kidney disease and progression; Mineral metabolism and bone diseases; Clinical nephrology, epidemiology, dialysis, and kidney transplantation. Editors only encourage the submission of valuable research to convey important advances in the field of nephrology (**Table 3**).

Additionally, a co-citation analysis identified the highly cited, influential studies in this subject. KIDNEY INT had the most citations (2741), the official journal of the International Society of Nephrology. KI is one of the most highly cited journals in the field of nephrology and is widely recognized as a top-tier journal worldwide for advancements and consequences related to kidney diseases (**Figure 5**). Therefore, it is appropriate to view the initial laboratory and clinical studies on complex pathology of diabetic nephropathy that have been published in this journal as landmark studies. To publish a manuscript in the KIDNEY INT will be a significant task for researchers. Nephrology has the highest percentage of papers (86.83%), making it the most well-represented research field. Co-authorship and co-citation analyses in writers may show possible collaborators and promising authors in this field (**Figure 5b**).

Kretzler, matthias and Saleem, moin a. are both among the top ten most active and cited authors (**Table 5**). The high concentration of co cited authors, including Mundel p (527 citations) and Susztak k (466 citations), ensures the dependence and recognition of research data sources, enhancing the credibility and relevance of the study (**Figure 7**). Based on the top 10 most frequently cited articles (**Table 7**), the article titled “Research Progress on Mechanism of Podocyte Depletion in Diabetic Nephropathy,” published in the JOURNAL OF DIABETES RESEARCH, with Dai HR as the first author (2), suggests that diabetic nephropathy (DN) and glomerular hyperfiltration are considered inducers of microvascular complications in the early stages of diabetes (**Figure 8**). The increase in urine protein levels in DN may be related to functional and morphological changes in podocytes, primarily including podocyte hypertrophy, epithelial-mesenchymal transition (EMT), podocyte detachment, and podocyte apoptosis. This review focuses on novel molecular perspectives on podocyte injury during the progression of DN, which provide new therapeutic targets for the development of crucial renal protective therapies for DN in the next decade (5).

**Table 7:** Co-citation table of literature.

Rank	Title	Journal	Author(s)	Total citations
1	Research Progress on Mechanism of Podocyte Depletion in Diabetic Nephropathy	*JOURNAL OF DIABETES RESEARCH*	Dai HR	89
2	Glucose-induced reactive oxygen species cause apoptosis of podocytes and podocyte depletion at the onset of diabetic nephropathy	*DIABETES*	Susztak K	88
3	mTORC1 activation in podocytes is a critical step in the development of diabetic nephropathy in mice	*JOURNAL OF CLINICAL INVESTIGATION*	Inoki K	84
4	Impaired Podocyte Autophagy Exacerbates Proteinuria in Diabetic Nephropathy	*DIABETES*	Tagawa A	80
5	Role of mTOR in podocyte function and diabetic nephropathy in humans and mice	*JOURNAL OF CLINICAL INVESTIGATION*	Godel M	78
6	Diabetic Kidney Disease Challenges, Progress, and Possibilities	*CLINICAL JOURNAL OF THE AMERICAN SOCIETY OF NEPHROLOGY*	Alicic RZ	78
7	Molecular mechanisms of diabetic kidney disease	*JOURNAL OF CLINICAL INVESTIGATION*	Reidy K	67
8	From the periphery of the glomerular capillary wall toward the center of disease - Podocyte injury comes of age in diabetic nephropathy	*DIABETES*	Wolf G	65
9	Update on Diabetic Nephropathy: Core Curriculum 2018	*AMERICAN JOURNAL OF KIDNEY DISEASES*	Umanath K	60
10	The podocyte’s response to injury: Role in proteinuria and glomerulosclerosis	*KIDNEY INTERNATIONAL*	Shankland SJ	57

**Figure 11:**
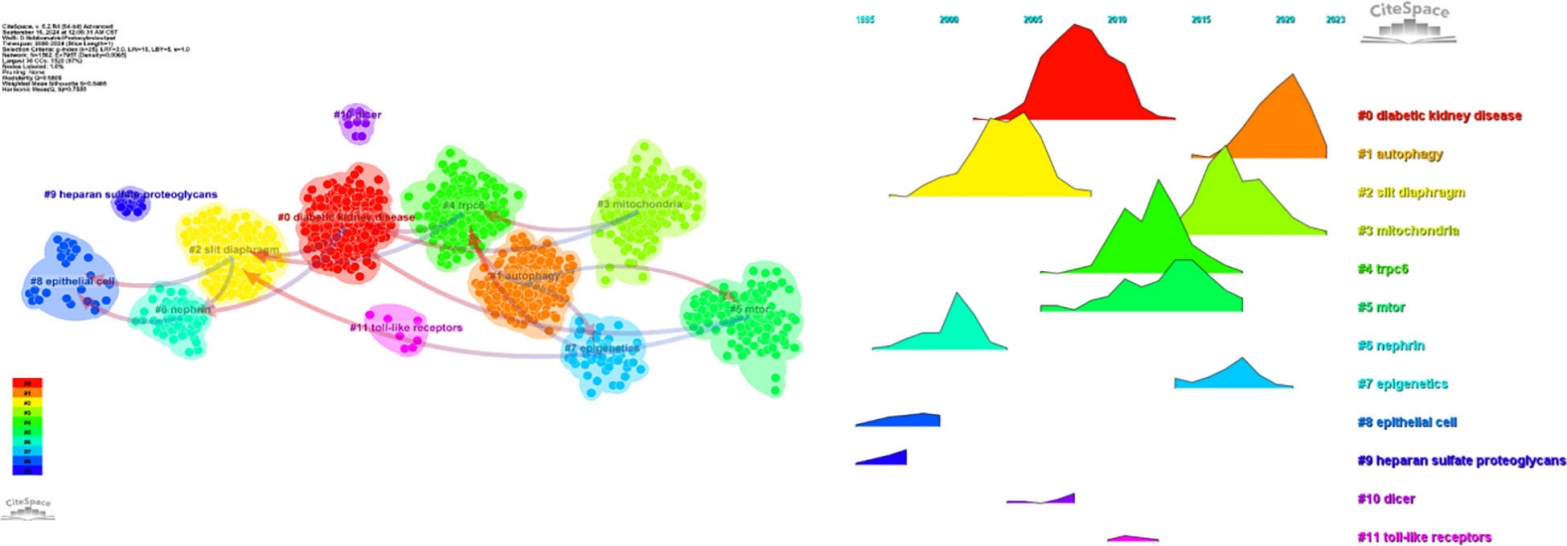
**(a)** Clustering of co-cited literature. **(b)** Peak map of co-cited literature.

Regarding the correlation between diabetic nephropathy and podocytes, a clustering analysis of the references cited in articles published over the past two decades reveals a trend in evolving hot topics in this field. From early topics such as diabetic kidney disease (cluster 0), slit diaphragm (cluster 2), nephrin (cluster 5), epithelial cell (cluster 8), and heparan sulfate proteoglycans (cluster 9), to mid-stage topics like rpc6 (cluster 4), mtor (also in cluster 5), dicer (cluster 10), and toll-like receptor (cluster 11), and finally to later-stage topics including autophagy (cluster 1), mitochondria (cluster 3), and epigenetics (cluster 7), the changing trends in hot topics represent the continuous deepening of research into the cellular and molecular biological changes in podocytes associated with diabetic nephropathy (**Figure 11**).

### The analysis of hotspots and emerging topics

Keywords can reflect the research hotspots and directions in a specific field. From **Table 6**, the top 20 keywords appear more than 300 times. These keywords represent the research hotspots in the field of podocyte depletion in Diabetic Nephropathy. The more representative keywords include injury, apoptosis, oxidative stress, proteinuria(374) and inflammation(301) (**Table 6**, **Figure 9**). From these keywords, we can summarize the general situation of podocyte-depletion-related fields, including a. Podocyte apoptosis is a significant mode of podocyte injury in DN, closely associated with the production of proteinuria and the exacerbation of renal damage;b. Enhanced oxidative stress responses in a high-glucose environment induce podocyte apoptosis and injury, serving as an important pathogenic mechanism in DN; c. Podocyte damage disrupts the integrity of the glomerular filtration barrier, resulting in the excretion of large amounts of protein in the urine, known as proteinuria, which is a key clinical manifestation of DN; e. High glucose levels and oxidative stress induce local inflammatory responses in the kidney, further exacerbating podocyte injury and accelerating the progression of DN.

The density map of these keywords can show the high-frequency keywords in this field more intuitively (**Figure 9b**).

The network clustering analysis of keywords (totally divided into 6 clusters) can intuitively show this field’s research direction and scope. As shown in **Figure 9a**, we get 6 clusters. The keywords of cluster 1 (red) are mainly about the research of injury, apoptosis and oxidative stress in DN podocyte. The keywords of cluster 2 (green) are mainly about the research of progression, inflammation, diabetes, renal injury, insulin, deficiency, blood pressure, complication, model, macrophage, risk, streptozotocin, system, therapy, obesity. The keywords of cluster 3 (blue) are mainly nephrin, proteinuria, protein, glomerulosclerosis, growth factor, pedpcalyxin, epithelial cells, glomerulus, iga nephropathy, messenger rna, mutation, number, podocin, slit diaphragm. The keywords of cluster 4 (yellow) are mainly related to inhibition, pathogenesis, kinase, biology, receptor, glucose, rage, vegf, beta, angiogenesis, induction, nitric oxide, protein kinase c, mouse, damage. The keywords of cluster 5 (purple) may be the study of mesangial cells, renal fibrosis, down regulation, microrna, proliferation, biomarker, acute kidney injury, cancer, high glucose, integrin linked kinase, nf kappa b, tgf beta, up regulation (**Figure 9**).

These six clusters represent DN and podocyte research focus and scope to some extent (**Figure 10**). In addition, from this ranking, we can see that podocyte research in molecular immunology has become one of the critical research focuses (6). The biopsy technology of the kidney has been more widely applied in clinical practice, and microscopic research on podocytes can no longer meet the needs of researchers (7). The timeline viewer keywords can help us see the time of a topic in this field and help us explore this field’s evolutionary trajectory.

References with intense citation bursts refer to the sudden increase of citations of certain documents in a certain period, which can help us find emerging topics and research topics that have attracted much attention in a certain field (8).

**Figure 12:**
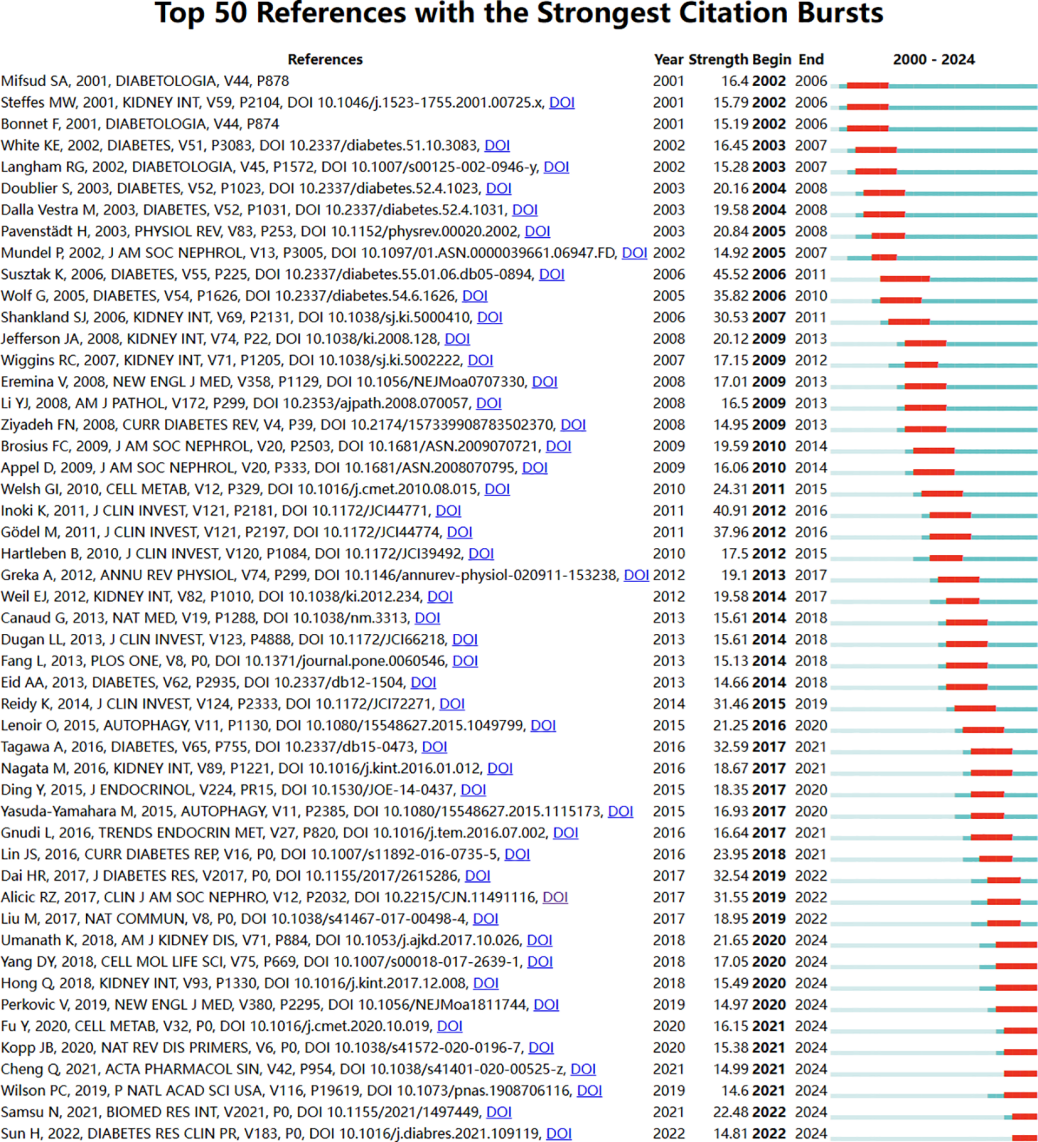
Bursting map of cited literature.

**Figure 13:**
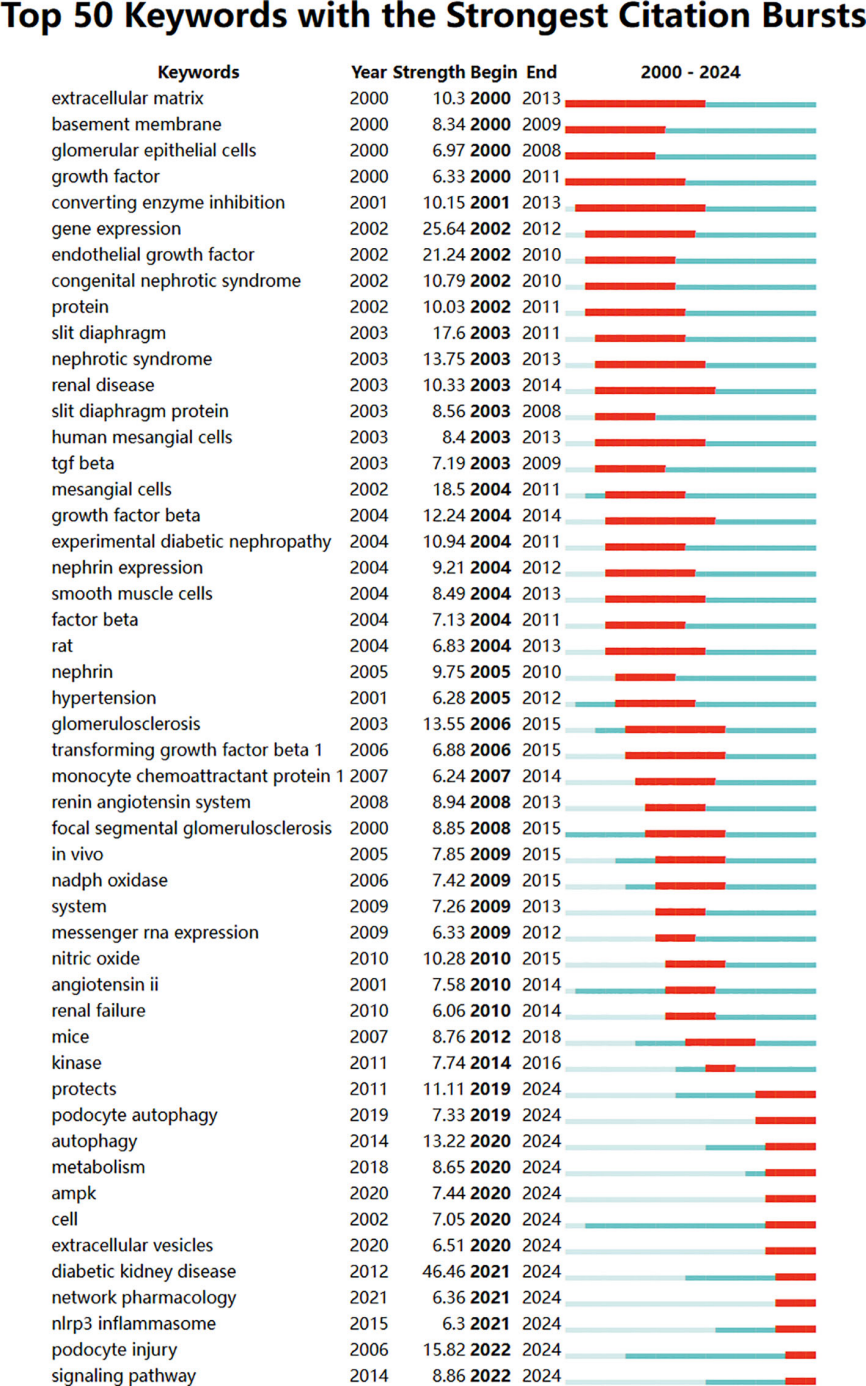
Bursting map of keywords.

This study obtained 465 references with the most powerful citation bursts and selected the top 50 among them (**Figure 12**). The paper “Glucose-induced reactive oxygen species cause apoptosis of podocytes and podocyte depletion at the onset of diabetic nephropathy” (Strength: 45.52) with the strongest citation burstness was a animal study published by Sustak K et al. in Diabetes in 2006 (9). Before that, people still knew little about the causes and consequences of podocyte loss in the early stage of diabetes nephropathy. This high-quality article observed that with the occurrence of hyperglycemia, podocyte apoptosis in the kidney increased sharply by intervening the feeding environment of Ins2 (Akita) mice with type 1 diabetes and Lepr (db/db) (db/db) mice with obesity and type 2 diabetes. This research result proves for the first time that ROS induced by glucose *in vivo* and *in vitro* can lead to podocyte apoptosis and podocyte depletion, and indicates that podocyte apoptosis/depletion is a new early pathological mechanism leading to diabetes nephropathy in the mouse model of type 1 and type 2 diabetes (10). This article believes that podocyte loss is an early feature of diabetes nephropathy and can predict its progression. This conclusion greatly boosted the confidence of subsequent researchers. Up to now, 18 papers (36%) in the top 50 are still in a state of citation burst, and the citation burstness of 13 papers has lasted for 3 years (years: 2020-2024). These 13 papers represent the latest research topics related to podocytes (**Figure 13**). According to a Ranking by burstness strength (from high to low), the No.1 paper (strength: 21.65) was published by Umanath K et al. in AM J KIDNEY DIS in 2018 (11). This Core Curriculum outlines and discusses in detail the epidemiology, pathophysiology, diagnosis, and management of diabetic nephropathy. It laid a foundation for the follow-up study of diabetes nephropathy and podocyte. Although this article did not directly elucidate the mechanism of podocyte injury, podocytes, as one of the endothelial cells of the glomerulus, are considered to have a similar mechanism to other arterial wall hyalinization, which is related to hyperglycemia and oxidative stress (12–14). Furthermore, the single best evidence-based therapy for diabetic nephropathy is therapy with a RAS-blocking medication (15). The second-ranked paper (strength: 17.05) was published by Yang DY et al. in The CELL MOL LIFE SCI in 2018 (16). This article deeply discusses the role of podocytes in diabetes nephropathy, especially the role of autophagy mechanism in podocyte injury and repair. Research shows that the imbalance of podocyte autophagy is one of the key factors leading to the progress of diabetes nephropathy. Therefore, the recovery of podocyte autophagy may become a new target for the treatment of diabetes nephropathy in the future (17). The third-ranked paper (strength: 15.49) was also published by Hong Q et al. in KIDNEY INT in 2018 (18). This article observed the effects of hyperglycemia, the role of SIRT1 in podocyte protection, and the therapeutic potential of BF175 as a SIRT1 agonist, revealing the key role of SIRT1 in podocyte protection and the potential application of BF175 as a SIRT1 agonist in the treatment of DKD. These findings provide important evidence for a deeper understanding of the mechanisms of podocyte injury and the development of new treatment methods (11, 19). The fourth-ranked paper (strength: 14.41) was published by Tung CW et al. in NEPHROLOGY in 2018 (20). In this paper, Wnt signaling pathway, Notch-1 signaling pathway and abnormal regulation of HDAC4 and miR-29a all play an important role in podocyte injury in diabetes nephropathy. The abnormal regulation of these signaling pathways not only leads to the destruction of podocyte structure and function, but also aggravates the pathological process of diabetes nephropathy (21).

The fifth-ranked paper (strength: 14.13) was published by Jin J et al. in STEM CELL RES THER in 2019 (22). This review delves into the mechanisms of podocyte injury in DKD, including the roles of mitochondrial dysfunction, lipid metabolism disorders, and the protective effects of key molecules such as ABCA1. At the same time, treatment approaches for podocyte injury have also been proposed, including drug intervention (such as drugs that activate ABCA1 expression) and gene therapy (such as gene therapy that increases ABCA1 gene expression), which may become new approaches for treating DKD. In addition, regulating signaling pathways under high glucose conditions (such as mTOR, Smad1, etc.) may also provide new ideas for the treatment of DKD (23).

The sixth-ranked paper (strength:14.04) was published by Garg Pet al. in AM J NEPHROL in 2018 (24). Foot injury usually manifests as fusion or disappearance of foot processes, which may lead to exposure of GBM and onset of glomerulosclerosis. The injury mechanism involves multiple factors, including immune response, toxin exposure, infection, ischemia, etc., which may lead to a decrease in the number of mature podocytes working normally (25). The phosphorylation status of Nephrin plays an important role in podocyte injury, and phosphorylation changes can affect the structure and function of podocytes (26). There are signaling pathways that interact between podocytes and glomerular endothelial cells, such as the endothelin-1 (EDN1)/endothelin receptor type A (EDNRA) signaling pathway, which may lead to mitochondrial oxidative stress and dysfunction in endothelial cells.

The seventh-ranked paper (strength: 13.68) was published by Anders HJ et al. in NAT REV NEPHROL in 2018 (27). This review argues that factors such as high blood sugar, hemodynamic changes, genetic factors, immune-mediated inflammation, and endothelial cell dysfunction work together to cause podocyte damage. Future treatment directions include treatment for high blood sugar, improvement of hemodynamics, anti-inflammatory and antioxidant therapy (13), podocyte protection therapy, as well as comprehensive treatment and personalized management. It also mentions the benefits of the drug SGLT2 on the kidneys. The eighth-ranked (strength:13.32) was published by Kato M et al. in NAT REV NEPHROL in 2019 (28). This review mainly explored that ln cRNAs, miRNAs, and epigenetic mechanisms play important roles in regulating podocyte gene expression and maintaining its function (29). In addition, metabolic memory phenomena may also persist in podocytes, leading to persistent podocyte dysfunction.

The ninth-ranked paper (strength: 12.92) was published by Ducasa GM et al. in J CLIN INVEST in 2019 (1). This review delves into the mechanisms of podocyte injury in DKD, including the roles of mitochondrial dysfunction, lipid metabolism disorders, and the protective effects of key molecules such as ABCA1. At the same time, treatment approaches for podocyte injury have also been proposed, including drug intervention (such as drugs that activate ABCA1 expression) and gene therapy (such as gene therapy that increases ABCA1 gene expression), which may become new approaches for treating DKD. In addition, regulating signaling pathways under high glucose conditions (such as mTOR, Smad1, etc.) may also provide new ideas for the treatment of DKD (30, 31).

And the tenth-ranked paper (strength: 12.01) was published by Brinkkoetter PT et al. in CELL REP in 2019 (32). This study reveals the severe impact of mitochondrial dysfunction on podocytes and the importance of TFAM genes in maintaining mitochondrial function.

The eleventh-ranked paper (Strength:11.63) was published by Forbes JM et al. in The NAT REV NEPHROL in 2018 (30). The study agrees podocytes damage mechanisms involve multiple aspects such as high blood sugar, oxidative stress, and abnormal fatty acid metabolism. The twelfth-ranked paper (strength: 11.11) was published by Fan YQ et al. in INT J BIOL SCI in 2019 (33). The study finds the mechanism of podocyte injury under high glucose conditions and the protective role of Sirt6 protein in it. Upregulation of Sirt6 expression or activation of its downstream signaling pathway may be a new approach (23).

Based on the above analysis and the related examination of the annexes, the following important insights can be derived: a. The critical role of podocyte function: Podocytes are a vital component in maintaining the integrity of the glomerular filtration barrier. b. The impact of a high-sugar environment on podocytes: It causes mitochondrial dysfunction and apoptosis, while also inducing oxidative stress and mitochondrial morphological abnormalities. c. The function of SIRT1 and SIRT6 proteins: They inhibit high glucose-induced mitochondrial dysfunction and apoptosis in podocytes by activating AMPK, thereby exerting protective effects (21, 34). d. The significance of SIRT1 in podocyte protection and the potential of BF175 as a novel SIRT1 agonist in the treatment of diabetic kidney disease (35). e. ADSCs-Exo can significantly reduce the apoptosis rate of MPC5 cells under high glucose conditions. Furthermore, immunoblotting experiments have revealed that ADSCs-Exo can decrease the activity of cleaved caspase-3 (30). Furthermore, from the cluster analysis of keywords (**Figure 10b**), it can be seen that autophagy plays an important role in the field of podocyte research.

## Strengths and limitations

This bibliometric analysis is the first to look at the development of research on podocyte injury and implications for diabetic nephropathy. Through investigations of co-authorship, co-citation, cooccurrence, and citation burst, we constructed and visualized the bibliometric networks using 2 well-known scientometric software tools (VOSviewer and CiteSpace). However, this study does have certain shortcomings. First of all, less quantitative analysis is used in this study and more qualitative analysis. Second, the WoSCC database was mostly used for the searches. The outcomes would be improved if they were coupled with data from other databases, such Scopus and PubMed. But it should be highlighted that WoSCC is the most popular scientometrics database.

## Conclusion

The findings presented herein may offer potential therapeutic targets and strategies for the management of DN associated with podocyte injury (36).

Notably, the widely recognized SGLT2 (sodium-glucose cotransporter 2) inhibitor, dapagliflozin, has demonstrated glycemia-independent renoprotective effects in a model of albumin overload (37). These protective effects extend to podocyte function, preventing dysfunction and loss due to its impact on mitochondrial protection (38). This interplay between podocyte research and the clinical treatment of diabetic nephropathy serves as a testament to their mutual reinforcement and support.

In the past 20 years, the research results on podocyte injury have increased year by year. This article provides some insights into the evolution of podocyte and the progress of DN (15, 38). This study assists scholars in locating collaborators and significant literature, provides guidance for publishing journals, and identifies research hotspots. This analysis acknowledges serves as a valuable resource for scholars and researchers, promoting continued exploration and collaboration in this critical area of study.

By examining the current state of the literature, we have uncovered several critical scientific and clinical gaps that demand further investigation. In the realm of scientific research, despite the notable progress we’ve made in elucidating the molecular mechanisms underlying podocyte injury, the intricate interplay among different signaling pathways and the role of epigenetic modifications remain largely obscure. Future research efforts should concentrate on utilizing cutting - edge technologies like single - cell RNA sequencing and multi - omics approaches to unravel these complexities. By doing so, we can identify novel therapeutic targets that allow for the specific modulation of podocyte function and survival.

From a clinical treatment perspective, the current therapeutic options for diabetic nephropathy (DN) - related podocyte injury are quite limited, primarily relying on glycemic and blood pressure control. There is an urgent need to develop targeted therapies that can directly protect podocytes and promote their regeneration. This could potentially involve repurposing existing drugs or discovering new small - molecule inhibitors or biologics based on the insights gained from basic research.

Moreover, genetic factors play a pivotal role in the pathogenesis of DN and podocyte injury. Studies have demonstrated that certain genetic variations can predispose individuals to an increased risk of developing DN. For instance, polymorphisms in genes encoding for proteins involved in podocyte structure and function, such as nephrin, podocin, and CD2 - associated protein (CD2AP), have been associated with DN susceptibility. These genetic variants may disrupt the normal architecture and function of podocytes, making them more vulnerable to damage in the setting of diabetes.

Genetic analysis has provided valuable insights into the complex interplay between genetic predisposition and environmental factors in DN development. Through genome - wide association studies (GWAS) and candidate gene studies, researchers have identified multiple genetic loci linked to DN. These findings not only enhance our understanding of the disease mechanisms but also offer potential targets for therapeutic intervention.

For example, if a specific genetic variant is found to be strongly associated with podocyte injury in DN, drugs could be developed to target the downstream signaling pathways affected by that variant. This personalized approach to treatment could lead to more effective therapies tailored to an individual’s genetic profile.

In addition to the need for targeted therapies, the early diagnosis of podocyte injury still poses a significant challenge. Large - scale, multicenter clinical trials are crucial for validating the sensitivity and specificity of proposed biomarkers. Genetic biomarkers, along with other traditional and novel biomarkers, could potentially be integrated into diagnostic panels to improve the early detection of podocyte injury.

The ultimate goal is to develop non - invasive diagnostic tools that can facilitate early intervention and improve patient outcomes. By combining genetic analysis with other research approaches, we can gain a more comprehensive understanding of the pathogenic factors of podocyte injury in DN. This holistic view will enable us to develop more effective prevention strategies, early diagnostic methods, and targeted treatments, ultimately transforming the clinical management of DN and improving the quality of life for patients.

In conclusion, the findings of this study represent a significant milestone in the field of podocyte research and its application to the clinical management of DN. However, they also highlight the need for sustained and targeted research efforts to address the existing gaps and unlock the full potential of podocyte - targeted therapies. Through interdisciplinary collaboration and a commitment to innovation, we can look forward to a future where DN - related podocyte injury is no longer a formidable challenge but a manageable condition with improved therapeutic options and patient outcomes.

## Data Availability

The datasets presented in this study can be found in online repositories. The names of the repository/repositories and accession number(s) can be found in the article/supplementary material.
